# A Common Regulatory Architecture Shapes Queen Chemical Signals Across Multiple Exocrine Sites

**DOI:** 10.1007/s10886-026-01750-2

**Published:** 2026-08-14

**Authors:** Margarita Orlova, Etya Amsalem

**Affiliations:** 1 Department of Entomology, Pennsylvania State University, University Park, PA 16802 USA; 2 Present Address: Department of Biology and Chemistry, SUNY Polytechnic Institute, Utica, NY 13502 USA

**Keywords:** Pheromones, Social insects, Glands, CHCs, Reproduction, Bumblebees

## Abstract

Insects communicate identity, health, and reproductive state through diverse chemicals secreted from multiple glands and production sites. Although these exocrine sites often contain a similar profile of compounds, they are usually studied in isolation. Here, we tested the hypothesis that changes in the identity and abundance of chemical signals from different exocrine sites within the same individual are coordinated, suggesting they may be governed by shared regulatory processes. To do so, we quantified the cuticular lipids and the glandular secretion of three exocrine glands in bumble bee queens across three key life stages: unmated newly-emerged queen (gynes), young founders shortly after nest initiation and old founders towards the end of the life cycle. We found that as queens aged and transitioned into reproduction, they produced hydrocarbons and esters with shorter chain length and shifted toward a greater investment in alkenes over alkanes across several exocrine sites. Terpenoid-, acetate- and wax-esters were produced across multiple exocrine sites with partially overlapping patterns. These remarkably coordinated shifts across glands that differ in both function and anatomical origin suggest that the overall chemical profile of an insect is coordinated across glandular sites and may convey more biologically relevant information than any single glandular secretion alone. Insect chemical communication is likely shaped by a shared regulatory architecture or coordinated responses to environmental conditions, highlighting a unifying mechanism underlying complex social signals.

**Supplementary Information:**

The online version contains supplementary material available at https://doi.org/10.1007/s10886-026-01750-2.

## Introduction

Insects possess multiple exocrine glands responsible for producing or storing compounds with both functional and signaling roles (Blomquist and Ginzel 2021). These glands receive particular attention in social insects, which have mastered the use of chemical signals to regulate nearly every aspect of sociality and show a marked expansion of abdominal and intersegmental glands (Billen and Šobotník 2015; Billen 2011). Most insects possess approximately 10–15 exocrine glands located in the head, thorax, abdomen, and beneath the cuticle, whereas social species such as bees, termites, and especially ants, may have up to 40 distinct glands (Billen and Morgan 1998). Studies about insect exocrine glands provided important insights into the information conveyed by individual secretions. Such information can be complementary; in the honey bee, for example, esters in the Dufour’s gland are associated with ovarian activation, differentially hydroxylated fatty acids in the mandibular glands with female caste, and phenolic compounds in the mandibular glands with queen mating status (Dor et al. 2005; Malka et al. 2009; Plettner et al. 1997). Signals across glands may also be redundant (Katzav-Gozansky et al. 2002) or even conflicting, requiring receivers to develop strategies for resolving them (Cournault and de Biseau 2009; Moore and Liebig 2010). An individual’s chemical profile emerges from the combined activity of multiple glands, yet glandular secretions are typically studied in isolation, overlooking their broader chemical context.

Gland secretions of insects are complex multicomponent mixtures containing diverse compounds such as amines, alkaloids, terpenoids, and pyrazines, but also broadly shared compounds across glands, particularly lipids. Lipids include multiple chemical classes such as hydrocarbons, fatty acids, alcohols, aldehydes, ketones, esters, and terpenoids. Within the same species, different glands often contain similar types of lipids. For example, similar hydrocarbons and wax esters are found in the Dufour’s and tergal glands of the honeybee (Katzav-Gozansky et al. 1997b; Wossler et al. 2000), whereas the Dufour’s, mandibular and metapleural glands of ants contain similarly structured free fatty acids, terpenes and ketones (Attygalle and Morgan 1984; Cammaerts et al. 1981a, b; Chen 2024; Vieira et al. 2012). Hydrocarbons often overlap substantially between exocrine sites (Mitra and Gadagkar 2014; Soroker et al. 1994), whereas other chemical classes, such as esters, show only partial overlap (Dietemann et al. 2003; Katzav-Gozansky et al. 1997b; Soroker and Hefetz 2000). Bumblebee females, for example, produce esters in both the Dufour’s and labial glands, but each gland contains a different family of esters that differ in their fatty acid moiety, with inversion between the ester family and their glandular site across two bumblebee species (Amsalem et al. 2009, 2014; Derstine et al. 2021; Orlova et al. 2022). More broadly, insects often produce lipids within a limited chain length range, likely reflecting biochemical constraints imposed by the fatty acid elongation machinery and downstream hydrocarbon synthesis pathways (Blomquist et al. 1987; Holze et al. 2021).

The glandular composition exhibits similarities and differences not only across glands, but it also varies dramatically across life stages. Across social insects, chemical profiles change predictably with age, reproductive status, mating, and social environment, reflecting shifts in physiological state and social role. Transitions associated with sexual maturation, mating, overwintering or diapause, colony founding and reproduction are often accompanied by changes in exocrine secretions, e.g., in queenless ants, the gamergate shifts her cuticular hydrocarbon profile after winning a dominance contest (Cuvillier-Hot et al. 2002), in honey bees queens, the profiles of the Dufour’s and mandibular glands change after mating (Nino et al. 2013; Slessor et al. 1990) and in bumble bee queens, the cuticular profiles change with age (Orlova et al. 2020). Thus, the glandular secretions are not static signals but plastic phenotypes that track life history stage and social context. The cooccurring changes in lipid compounds across life cycle and exocrine sites allude to their origin from common precursors via common pathways. However, it remains poorly understood whether changes across glands and across the life cycle are coordinated across exocrine sites and what mechanisms are responsible for such potential coordination.

Here, we examine the cooccurring changes in four sites of chemical secretion: the cuticular lipids (CL), the Dufour’s (DG), mandibular (MG), and labial glands (LG) in the same bumblebee queens (*Bombus impatiens*) across their life cycle (unmated gynes, young founders and old founders). Bumblebee queens are an excellent model system to examine cooccurring shifts in glandular secretions since their reproductive and social behavior changes dramatically within a short period. Queens emerge late in the season, then leave the colony, mate, and enter a 6–9 month winter diapause (Amsalem et al. 2015). In spring, queens found new colonies by laying and caring for the first brood until workers emerge. During this social phase, queens produce all eggs while workers remain sterile and perform nest duties. Colonies of *B. impatiens* can reach several hundred workers within a couple of months, after which workers begin laying unfertilized male eggs (Duchateau and Velthuis 1988), and the colony transitions to sexual production (gynes and males) and eventual queen death.

Several of the glands we focused on have been investigated previously and are known to serve different roles associated with queen maturity, ability to inhibit worker reproduction and regulate gyne production. The secretions of the Dufour’s and labial glands, as well as the cuticular secretion, change with age and reproductive status (Derstine et al. 2021; Orlova et al. 2022). The cuticular secretion of founder queens inhibits worker reproduction in a context-dependent manner when combined with eggs and applied to newly-emerged gynes (Orlova and Amsalem 2021). Secretions of the Dufour’s and labial glands, particularly their terpenoid esters and terpene components, are produced in greater quantities by gynes, and are involved in mating and sex pheromone signaling (Spence and Amsalem 2025). Finally, the mandibular glands, although not examined directly in *B. impatiens*, were studied in another bumblebee species (*B. terrestris*) that often serves as a point of comparison, and was initially suggested to inhibit worker reproduction (Honk van et al. 1980), a claim that was later refuted (Bloch and Hefetz 1999). More recently, the mandibular gland secretion was shown to regulate larval development duration, suggesting a role in female differentiation into workers or gynes (Franco et al. 2023). The composition and function of the mandibular glands in *B. impatiens* have not been investigated previously, but they may serve a similar function. Altogether, examining the co-occurring changes in compound abundance across these chemically and functionally distinct exocrine sources could reveal whether changes in secretion composition are coordinated across different signaling sites.

## Methods and Materials

### Bees

Colonies of *Bombus impatiens* (*n* = 16) were obtained from Koppert Biological Systems (Howell Michigan, USA) or reared in the lab from newly-emerged gynes. Colonies were maintained in the laboratory under constant darkness, a temperature of 28–30 °C, 60% relative humidity, supplied *ad libitum* with a 60% sucrose solution and fresh pollen (Koppert Biological Systems, Howell, Michigan, USA) and were used as a source for queens. All bees were reared under identical conditions and provided with the same diet throughout the study. We generated three groups of queens across their life cycle: (1) unmated gynes, (2) young founders, and (3) old founders. Unmated gynes (*n* = 14, 2–6 per colony, 4 colonies) were sampled from the colonies upon emergence, marked and returned to their natal colonies. Gynes were transferred into individual cages (11 cm x 7 cm) 3 days later and were frozen at the age of 6 days. Young founders (*n* = 16, 1–3 per colony, 8 colonies) were mated with unrelated males and treated with CO_2_ at the age of 6–11 days (Treanore et al. 2021), after which they were placed in small cages until the first worker emerged. CO₂ treatment is a common practice used to bypass diapause and induces reproduction and colony founding in bumblebee queens (Amsalem and Grozinger 2017; Barie et al. 2022). Young founders were sampled at the age of approximately 30 days. Old founders (*n* = 8, each from a unique colony) were obtained directly from old commercial colonies that were reared in the lab. These colonies contained more than 100 workers and were producing sexuals (gynes and males). These queens were approximately 90 days old or more at sampling. The queen groups were sampled nonlethally for cuticular lipids over three consecutive days prior to freezing (see: “cuticular lipid extraction” below). Frozen queens were dissected and their ovarian activation was measured (see “Ovary dissection” below). During dissection, we further extracted the Dufour’s, labial, and mandibular glands (see “Gland Dissection” below).

### Cuticular Lipid Extraction

Cuticular lipid (CL) samples were obtained from live queens by a non-lethal sampling method described in (Orlova et al. 2020). Samples were obtained for three consecutive days for each queen prior to lethal sampling. Briefly, each queen was placed in a sterile 20 mL glass vial for 10 min. After 10 min the queen was released back to her cage/colony and the vial was washed with 500 µL hexane. The resulting sample was then transferred to a 2 mL vial, evaporated to a volume of 200 µL and transferred into an insert. The resultant sample was evaporated completely and reconstituted with 50 µl hexane with 1 µg pentadecane (*n*-C15) as an internal standard. The vials were stored at -20 ˚C until analysis.

### Ovary Dissection

The content of the abdomen was thawed in a drop of distilled water. Ovaries were separated from other tissues and moved into a separate drop of water. They were dissected under a stereomicroscope and the largest three terminal oocytes across both ovaries (one at each ovary) were measured with an eyepiece micrometer. The mean length of these oocytes was used as a measure of ovary activation (Amsalem et al. 2009).

### Gland Dissection

The Dufour’s gland was separated from the oviduct and other abdominal tissues. The cephalic labial glands were dissected from the head capsule by opening the sclerotized cuticle with forceps and separating the two clusters of gland acini, small saclike cavities that form the glands, from surrounding tissues. The mandibular glands were obtained by opening the sclerotized cuticle around each mandible and separating the gland sacs from the mandibles. After dissection, the Dufour’s and labial glands were individually placed in glass vials containing 50 µL hexane with 1 µg pentadecane (*n*-C15) as an internal standard. Because the mandibular glands contained more polar compounds and large amounts of fatty acids that can obscure other compounds, they were placed in 50 µL methanol, evaporated to dryness, derivatized with 10 µl BSTFA, and incubated for 4 h at room temperature to complete the reaction, after which 50 µL hexane with 1 µg pentadecane (*n*-C15) as an internal standard was added to each sample. BSTFA derivatization converts fatty acids into their trimethylsilyl derivatives, improving peak shape, volatility, and chromatographic separation while preventing broad peaks from obscuring other compounds. All vials were stored at − 20 °C.

### Chemical Analysis

All samples were evaporated to a volume of 10 µL, of which 1 µL was run on a gas chromatograph for quantitative analyses. Samples were identified using an Agilent 7890 A GC equipped with a HP-5ms column (0.25 mm id x 30 m x 0.25 μm film thickness, Agilent, Santa Clara CA, USA) that was interfaced to an Agilent 5975 C mass selective detector operated in electron impact ionization mode (70 eV). The oven temperature was programed from 60˚C to 120˚C at 15˚C/min, then 4˚C/min to 300˚C (5 min hold). The injector port and FID were held at 250˚C and 320˚C, respectively. Compounds were identified based on diagnostic ions in the resulting spectra and retention indices relative to straight-chain alkanes. Samples were quantified using a Trace 1310 GC (Thermo Fisher, Waltham, MA, USA) equipped with a flame-ionization detector (FID) and a TG-5MS column (0.25 mm id x 30 m x 0.25 μm film thickness, Thermo Fisher). The GC oven temperature program and conditions were the same as above.

### Data Analysis of Glandular and Cuticular Secretions

Absolute amounts (in nanograms), relative amounts (as proportions of the total secretion) and retention indices were calculated for all compounds (Table S1A-D). Average chain length was computed by multiplying the number of carbons in each hydrocarbon by the share of that hydrocarbon in the hydrocarbon fraction and summing the products of this multiplication. Average chain length was also calculated for wax esters and acetate esters based on the sum of the acid and the alcohol moiety chain lengths. Given that multiple wax esters of the same molecular weight co-eluted and contributed to the same peak, it was impossible to calculate their relative amount, and, consequently, the average chain length of alcohol and acid moieties separately. GC/MS analysis of the mandibular gland samples detected large and highly variable amounts of palmitic, oleic and stearic acids, their methyl esters derived from incubation in methanol and their trimethylsilyl esters derived from silylation. For the purpose of subsequent analyses, the amounts of these acids and their derivatives were summed by chain length and treated as a single compound.

### Statistical Analysis

Statistical analyses of chemical composition of glands were performed using R Studio. Permutational multivariate analysis of variance (PERMANOVA, *adonis* and *adonis2* functions in R) were used to compare chemical profiles in their entirety between groups. To compare specific chemical properties across glands between queen types, we used pairwise permutational multivariate analysis of variance (*pairwise.perm.manova* function in R) with Wilks test and FDR adjustment for multiple comparisons. Pseudo-F and p-values for terms of interest are reported in the results. Random Forest analysis was used to determine specific components accounting for the difference between chemical profiles and replicated 20 times using VSURF algorithm to ensure that the results were consistent across replications (*RandomForest* and *VSURF* functions in R). Non-parametric Spearman correlation analysis (cor.test function in R) was used to analyze the correlation between chemical parameters across exocrine sources. Kruskal-Wallis (kruskalTest function in R) tests were performed on oocyte sizes, as well as proportions and average chain lengths of specific compounds within chemical profiles. Figures were generated using JMP 18. Statistical significance was accepted at α = 0.05.

## Results

### Oocyte Size

**Fig. 1:**
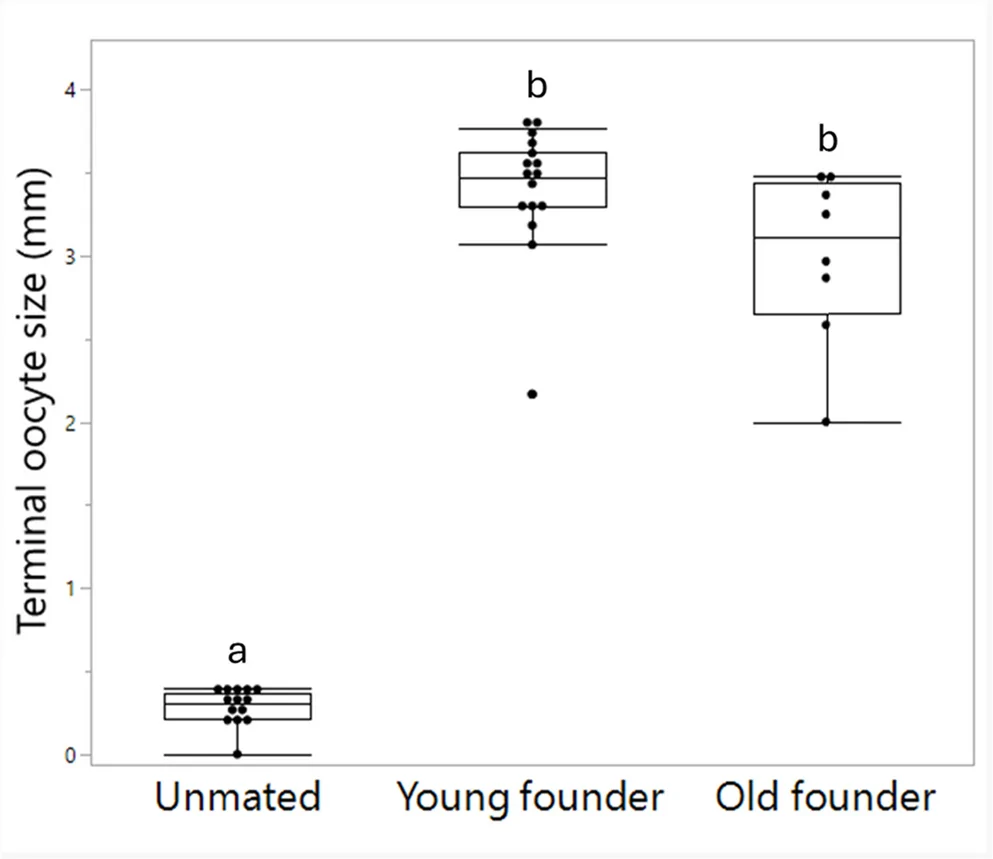
Ovary activation in queen groups. Both founder queen groups fully activated their ovaries whereas the unmated gynes had inactivated ovaries. Letters above columns represent statistical differences at α = 0.05

The average oocyte size was smaller in unmated gynes than in either founder, and no differences were detected between the two founders, both of which had fully activated ovaries (Kruskal-Wallis test, H_2_ = 27.85, *p* < 0.0001 followed up by Dunn’s test, *p* < 0.01 for unmated gynes vs. other types, *p* > 0.05 for young vs. old founders, Fig. 1).

### Chemical Profiles of Queens

**Fig. 2:**
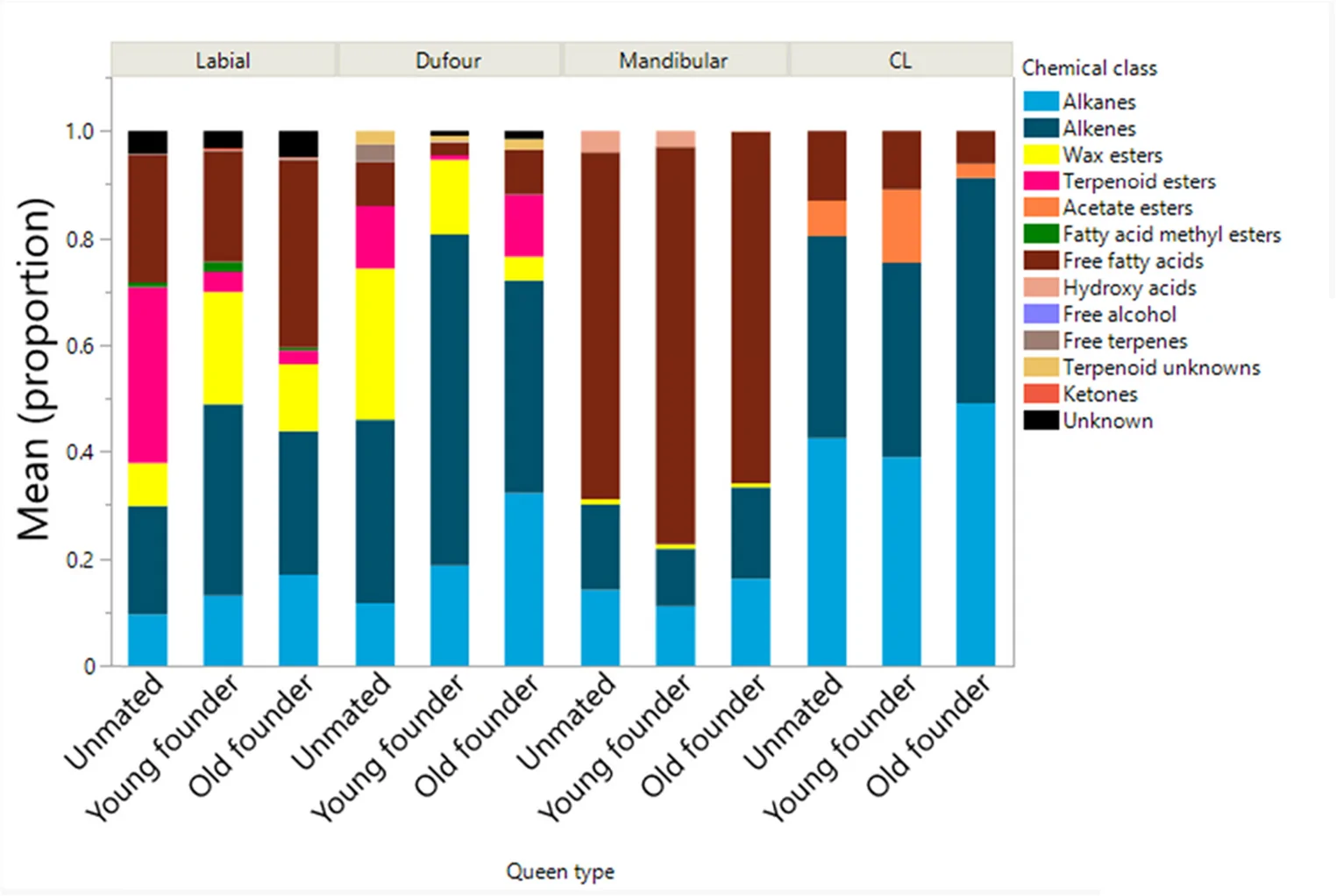
The proportion of compounds divided by chemical class across queen types (unmated, young and old founders) and exocrine sites (labial, Dufour’s and mandibular glands, and cuticular lipids)

The dominant compounds across all exocrine sites were hydrocarbons. The labial (LG) and Dufour’s glands (DG) contained also large amounts of wax and terpenoid esters and small amounts of terpenes. The cuticular lipids (CL) contained also acetate esters, and the mandibular glands (MG) contained large amounts of fatty acids. Additional chemical classes across the glands and the cuticle included small amounts of alcohol in the DG, ketones in the LG, and hydroxy acids in the MG (Table S1, Fig. 2).

**Fig. 3:**
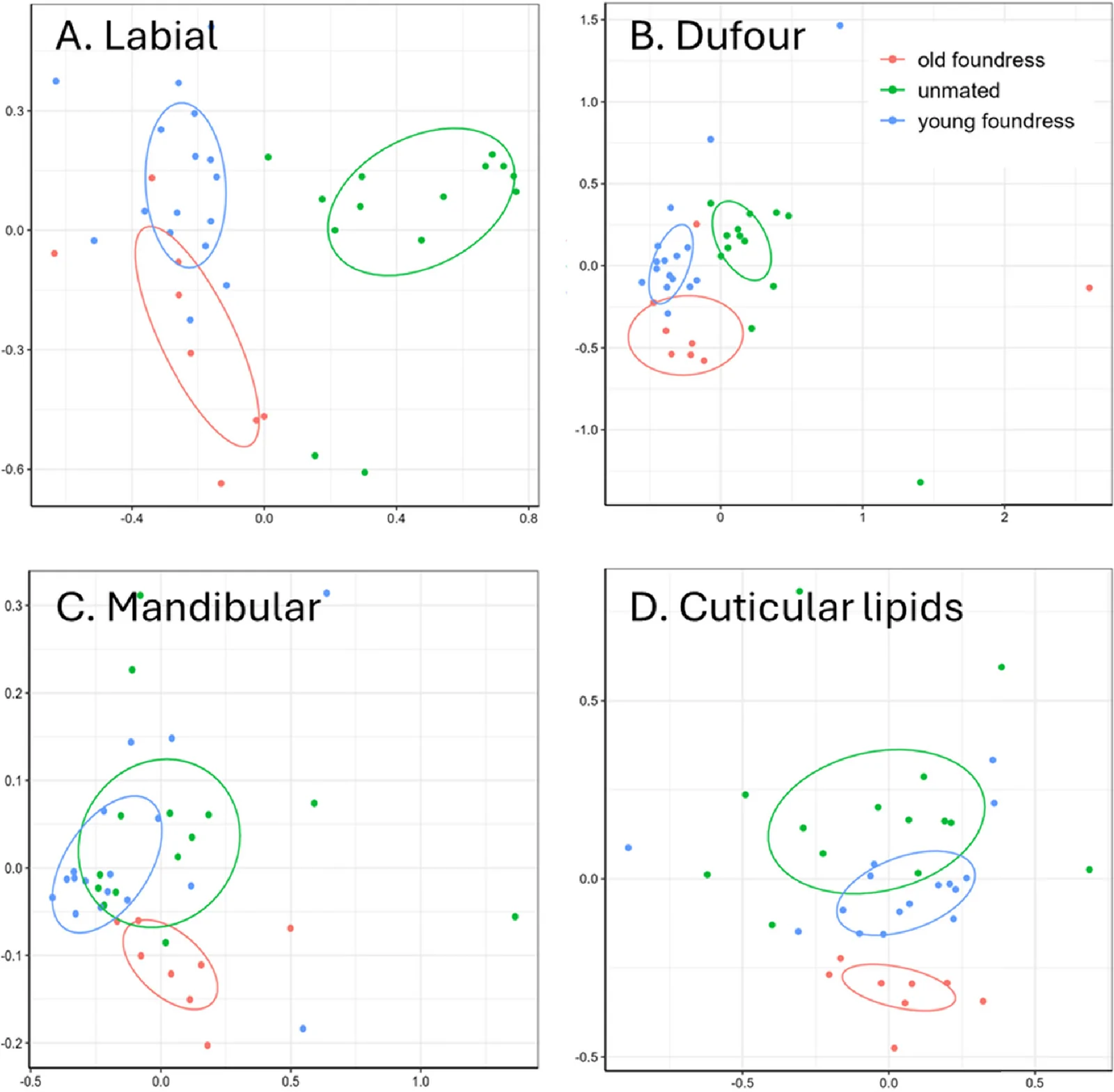
NMDS plot showing clustering of queen types (unmated, young and old founders) across exocrine sites (labial, Dufour’s and mandibular glands, and cuticular lipids)

NMDS analysis showed significant separation among queen types based on the entire chemical profiles of the LG, DG, and CL (Pseudo-F = 14.22, R^2^ = 0.45, *p* < 0.001, Pseudo-F = 6.69, R^2^ = 0.27, *p* < 0.001 and Pseudo-F = 5.33 R^2^ = 0.23, *p* < 0.001 for LG, DG, and CL, respectively, Fig. 3). There was no significant separation between queen types based on MG secretion (Pseudo-F = 2.05, R^2^ = 0.10, *p* = 0.08) due to large variability in the amounts of oleic and stearic acids and their derivatives that dominate the secretion. Removing these components from the analysis resulted in a significant difference between queen types (Pseudo-F = 2.8, R^2^ = 0.13, *p* = 0.01).

In all secretions, the top 5 compounds contributing to separation included hydrocarbons (Table S2). However, in the DG, LG and CL, the compounds with the highest contribution to profile separation were secretion-specific non-hydrocarbon components – wax esters in the LG, acetate esters in the CL and free terpenes in the DG.

### Hydrocarbon-Associated Shifts Across All Exocrine Sites

**Fig. 4:**
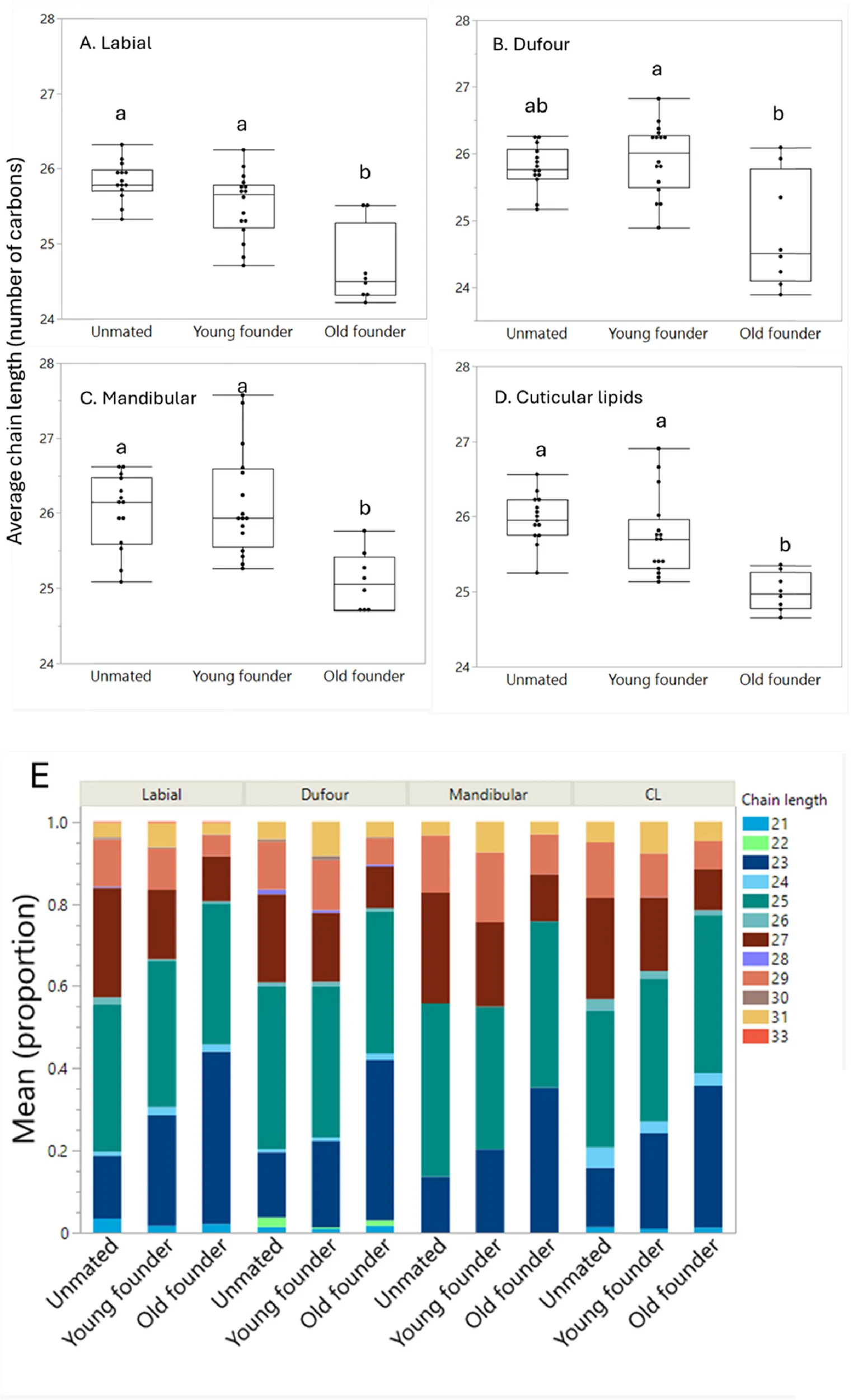
The average chain length of carbons (**A**-**D**) and the proportion of carbon chain length (**E**) across queen types (unmated, young and old founders) and exocrine sites. Letters above columns represent statistical differences at α = 0.05

The average chain length of hydrocarbons differed significantly between queen types across all secretions (Kruskal-Wallis test, H_2_ > 8.8, *p* < 0.05 for all comparisons, Fig. 4A-D), with significantly shorter average chain length in the LG, MG, and CL of old founders compared with the other queen types (pairwise Dunn’s test, *p* < 0.01 for old founders vs. other queen types, *p* > 0.05 for young founders vs. unmated gynes). A similar pattern was observed in the DG, but differences were detected only between old and young founders, with unmated gynes forming an intermediate group (pairwise Dunn’s test, *p* < 0.01 for old vs. young founders, *p* > 0.1 for other comparisons). The dominant carbon chain length across all exocrine sites was 25, with similar proportions across sites and little variation across life stages (Fig. 4E). Other dominant chain lengths were 23, 27, and 29, which varied notably throughout the life cycle, mainly with an increase in chain length 23 and a reduction in 27 and 29 as queens aged.

**Fig. 5:**
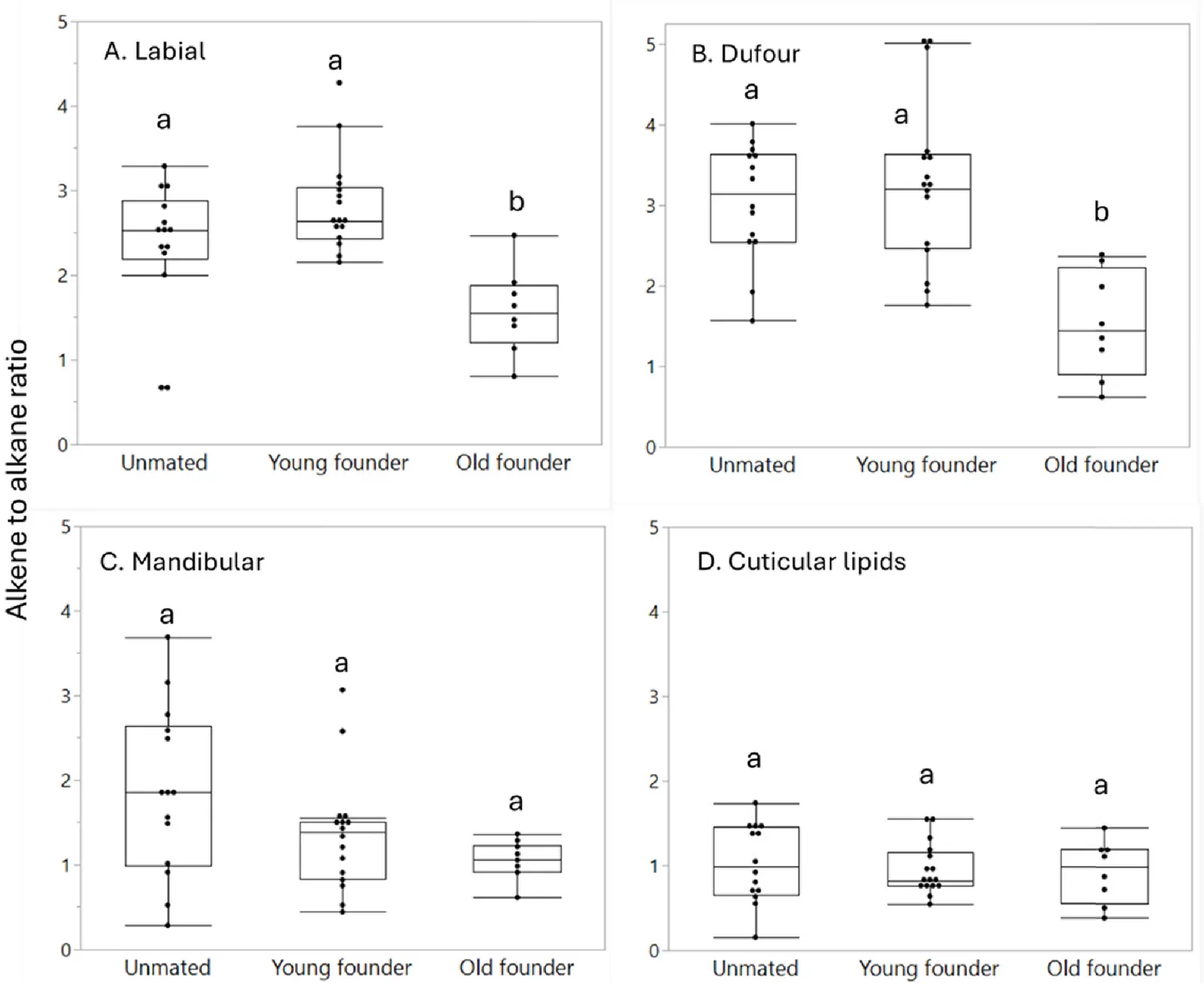
The alkene to alkane ratio across queen types (unmated, young and old founders) and exocrine sites (labial, Dufour’s and mandibular glands, and cuticular lipids). Letters above columns represent statistical differences at α = 0.05

The ratio of alkene (unsaturated hydrocarbons) to alkane (saturated hydrocarbons) was significantly lower in old founders than in other queen types in DG and LG (Kruskal-Wallis test, H_2_ > 14.1, *p* < 0.001 for both glands, pairwise Dunn’s test, *p* < 0.02 for old founders vs. other queen types, *p* > 0.05 for young founders vs. unmated gynes, Fig. 5). The same trend was observed in the MG, but the significance was marginal (Kruskal-Wallis test, H_2_ = 5.5, *p* = 0.06). No such difference was found in the CL.

### Esters-Associated Shifts in Exocrine Sites

Three types of esters were found across exocrine sites: terpenoid and wax esters, both found in the LG and DG, and acetate esters, found only on the cuticle. Terpenoid esters in the DG included farnesyl and geranylgeranyl esters of 16 and 18 carbon acids, found mostly in unmated gynes. In LG, farnesyl, dihydrofarnesyl, and geranyl esters of multiple even numbered fatty acids ranging from 12 to 18 carbons were detected. Dihydrofarnesyl and geranyl esters were present almost exclusively in unmated gynes (Kruskal-Wallis test, H_2_ > 24, *p* < 0.01 for unmated gynes vs. other queen types for both comparisons). Wax esters shorter than 30 carbons, mostly octyl and decyl esters of 16- and 18-carbon acids, were only found in LG, while esters of 30 carbons or longer were found in both LG and DG. Acetate esters were unique to CL and included tetracosyl, hexacosyl and octacosyl acetates.

**Fig. 6:**
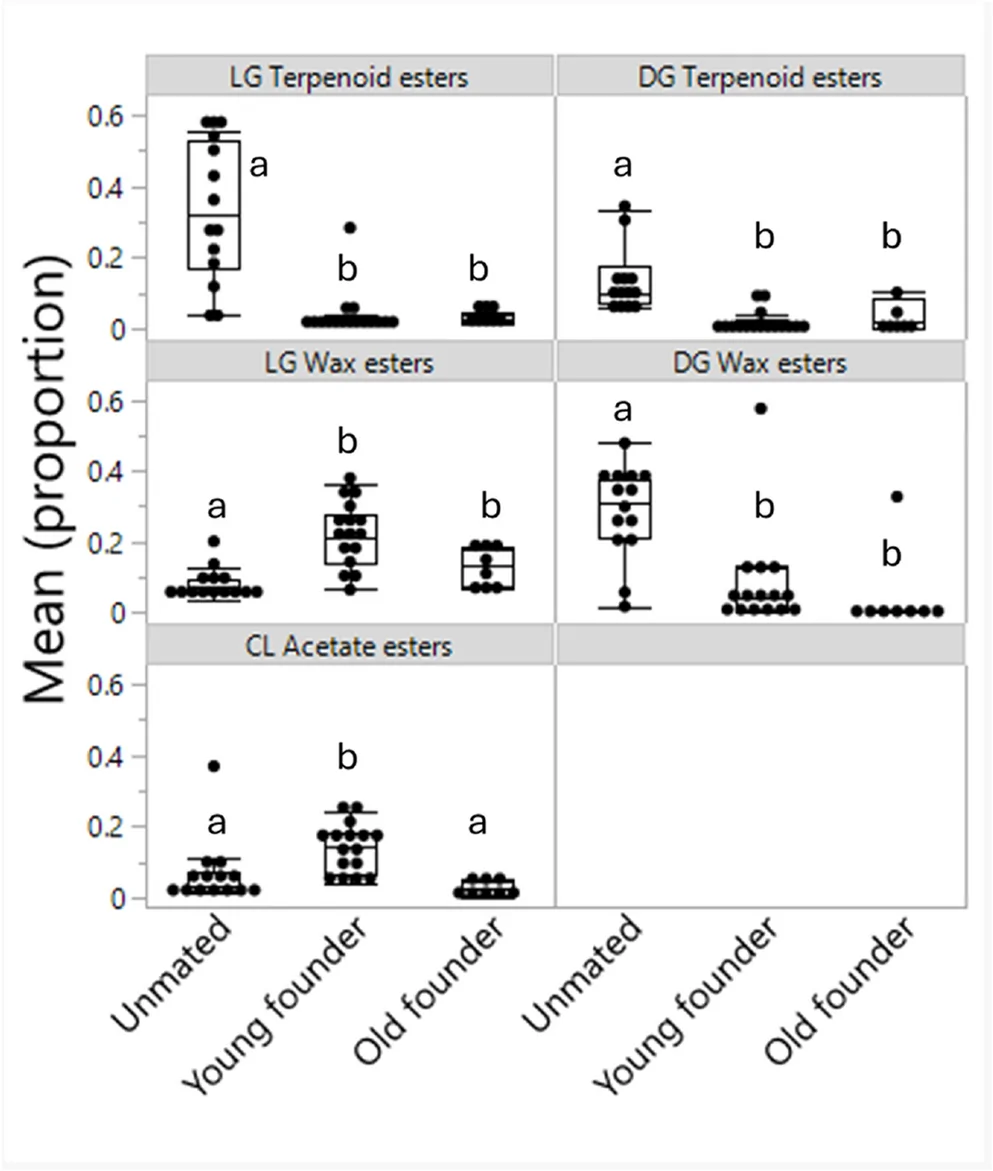
The proportion of terpenoid, wax and acetate esters in the labial (LG), Dufour’s glands (DG) and cuticular lipids (CL) across queen types (unmated, young and old founders). Letters above columns represent statistical differences at α = 0.05

The proportion of terpenoid compounds, mostly terpenoid esters, was significantly higher in unmated gynes than in the other two queen types in DG and LG (Kruskal-Wallis test, H_2_ > 17.3, *p* < 0.001 for both glands, pairwise Dunn’s test, *p* < 0.05 for unmated gynes vs. other queen types, *p* > 0.05 for old vs. young founders, Fig. 6). The proportion of wax esters in DG was highest in unmated gynes (Kruskal-Wallis test, H_2_ = 16, *p* < 0.001, pairwise Dunn’s test, *p* < 0.03 for unmated gynes vs. other queen types) with no difference between old and young founders (pairwise Dunn’s test, *p* > 0.2). In contrast, the proportion of wax esters in LG was lower in unmated gynes than in the other queen types, with no difference between old and young founders (Kruskal-Wallis test, H_2_ = 17.2, *p* < 0.001, pairwise Dunn’s test, *p* < 0.001 for unmated gynes vs. other queen types, *p* > 0.2 for young vs. old founders). The proportion of acetate esters in CL, as well as the proportion of tetracosyl acetate within the acetate fraction, were significantly higher in young founders than in the other queen types (Kruskal-Wallis test, H_2_ > 18, *p* < 0.001, pairwise Dunn’s test: *p* < 0.004 for young founders vs. other queen types, *p* > 0.5 for unmated gynes vs. old founders, Fig. 6).

**Fig. 7:**
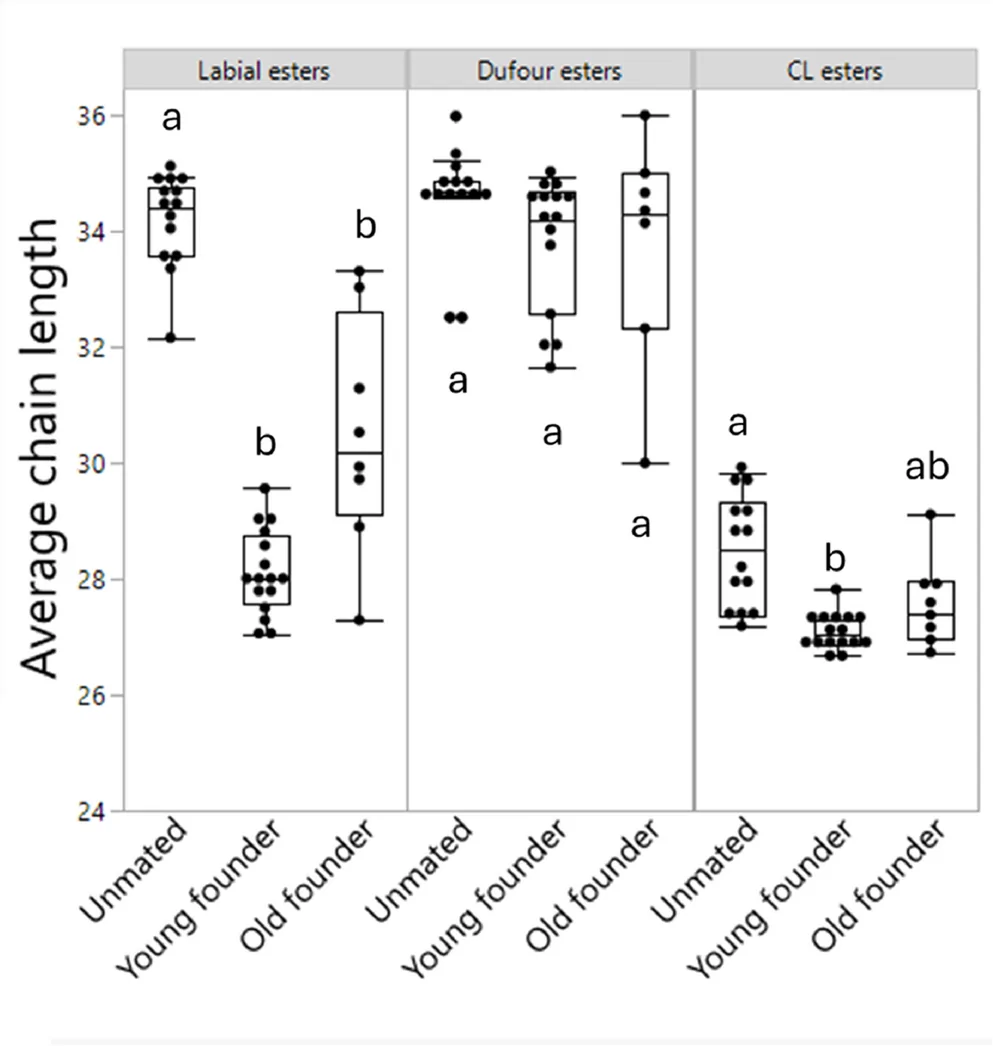
The average chain length of esters in the Dufour’s and labial glands and cuticular lipids across queen types (unmated, young and old founders). Letters above or below columns represent statistical differences at α = 0.05

The average chain length of LG wax esters and CL acetate esters, determined by the alcohol moiety, but not of DG wax esters, differed significantly among queen types (Kruskal-Wallis test, H_2_ = 28.8, *p* < 0.01, H_2_ = 2.3, *p* = 0.26 and H_2_ = 17.7, *p* < 0.01 for LG, DG and CL respectively, Fig. 7). In LG, unmated gynes had significantly longer esters than the two founders (pairwise Dunn’s test, *p* < 0.05 for unmated gynes vs. other queen types, *p* > 0.1 for young vs. old founders). In CL, unmated gynes had significantly longer acetate esters than young founders, with old founders forming an intermediate group (pairwise Dunn’s test, *p* < 0.001 for unmated gynes vs. young founders, *p* > 0.17 for other comparisons). This pattern was likely driven by the very high overall abundance of acetate esters in young founders, most of which consisted of tetracosyl acetate, the shortest compound in this class.

### Correlations Among Chemical Profile Metrics Across Exocrine Sites

The average hydrocarbon chain length across all secretions was significantly correlated (Spearman’s ρ > 0.5, *p* < 0.01 for all correlation analyses) and these correlations persisted after unmated gynes were excluded. Ratios of alkenes to alkanes were correlated among all three exocrine secretions (Spearman’s ρ > 0.4, *p* < 0.01) excluding CL, and these correlations persisted after removal of unmated gynes. The proportion of terpenoid compounds was significantly correlated between DG and LG (Spearman’s ρ = 0.52, *p* < 0.01, respectively). Average hydrocarbon chain length metrics across all secretions significantly separated the three queen types (Pseudo-F = 16.23, R^2^ = 0.48, *p* = 0.001, pairwise comparisons: *p* = 0.003 for old founders vs. other queen types, *p* = 0.007 for unmated gynes vs. young founders for average chain lengths). Alkene to alkane ratios across exocrine secretions separated old founders from the other queen types but did not differ between unmated gynes and young founders (Pseudo-F = 8.96, R^2^ = 0.33, *p* = 0.001, *p* = 0.0015 for old founders vs. other queen types, *p* = 0.19 for unmated gynes vs. young founders). The average chain lengths of LG wax esters and cuticular acetate esters were strongly correlated (Spearman’s ρ = 0.6, *p* < 0.001), but no correlation was found between the wax esters in DG and LG.

## Discussion

Our results indicate similar chemical shifts across some or all exocrine sites examined during the queen life cycle. As queens aged, they produced shorter hydrocarbons and esters and less unsaturated hydrocarbons. Terpenoid ester proportions decreased similarly after mating across secretions, while cuticular acetate esters and labial wax esters increased. Overall, secretions from different exocrine sources, most likely serving different functions, showed several coordinated changes, suggesting central regulation. Such central regulation could stem from functional similarity among glands, a shared site of synthesis from which compounds are transported to different exocrine locations, or common enzymatic machinery acting on a relatively narrow range of precursors shared between exocrine sources. Coordinated changes may also arise from shared responses to environmental conditions such as nutrition, temperature, and humidity, as well as sociobiological factors such as colony size. However, with the exception of colony size, these conditions were identical across our treatment groups. Moreover, even if such factors contribute to the observed patterns, they would still have to act through central regulatory mechanisms that coordinate changes across different exocrine sites.

While the signaling functions of individual compounds in *Bombus* are still poorly understood, what little is known about the behavioral activity of different secretions suggests a degree of functional overlap between exocrine sources and a certain redundancy of different signals. Both the Dufour’s and the labial gland secretions of gynes attract males in combination with visual and likely auditory signals (Spence and Amsalem 2025). Profiles of the labial, Dufour’s and cuticular secretions all correlate with reproductive status and apparently help workers identify potential helpers or, conversely, potential competitors (Amsalem and Hefetz 2010; Amsalem et al. 2009, 2014; Derstine et al. 2021; Orlova and Amsalem 2021; Orlova et al. 2020, 2022). Such similarities in both function and composition may suggest that different exocrine sources react in similar ways to large-scale physiological events such as changes in the titers of ovarian hormones or activation of the spermatheca and ovaries.

Alternatively, similar shifts in the content of different glands may reflect a common source from which compounds, or at least their precursors, arrive at different sites of secretion. Lipid biosynthesis in insects is relatively well studied and was shown to rely on a conserved and relatively limited set of enzymatic pathways (Blomquist and Bagnères 2010). Alkyl chains are formed from specific fatty acyl precursors by fatty acid synthases, lengthened by elongases or shortened through β-oxidation pathways. Double bonds are introduced by fatty acyl desaturases with defined positional and stereochemical specificities. Alcohols are produced through the reduction of fatty acyl intermediates by fatty acyl reductases and may be conjugated with a fatty acyl-CoA substrate by acyltransferases to form diverse esters or oxidized to aldehydes or back to acids by dehydrogenases and oxidases. Methyl branching patterns, characteristic of many social insect secretions, arise from the incorporation of methyl-malonyl CoA during fatty acid alkyl chain synthesis. Importantly, many of these reactions occur outside of exocrine glands themselves in tissues with extensive metabolic activity such as the fat body or oenocytes embedded in the epidermis, with final products or partially modified compounds subsequently transported to exocrine glands for storage, further modification, or release (Blomquist and Bagnères 2010). Thus, centrally regulated changes in the lipid output of the fat body would result in simultaneous shifts in chemical profiles across multiple exocrine sources.

The most striking pattern that we observed across all four exocrine sources we examined was the decrease of average hydrocarbon chain length with age in founder queens. This shift was driven by a transition from longer-chain hydrocarbons (mostly 27 and 29 carbons) toward shorter-chain hydrocarbons (mostly 23 carbons). While the regulation of hydrocarbon biosynthesis remains poorly understood in Hymenoptera, the underlying pathway has been studied in several Diptera species. In the housefly, ovarian development is accompanied by a shift from longer-chain hydrocarbons (27 carbons) to shorter-chain hydrocarbons (23 carbons) and is induced by increased levels of ovary-derived hydroxyecdysone (Adams et al. 1984). More recent works in Drosophila (Chiang et al. 2016; Dembeck et al. 2015) identified genes responsible for carbon chain elongation and found that hydrocarbons of 25 carbons or longer are produced as a cluster by a specific set of enzymes distinct from those producing hydrocarbons of 24 carbons or shorter. However, neither of these studies propose a physiological pathway activating this enzymatic machinery. Interestingly, a recent study in the Argentine ant *L. humile* (Moyneur et al. 2026) found that exposure to methoprene, a juvenile hormone analogue, also decreases the abundance of hydrocarbons of 25 carbons or longer, but not of those with shorter chain lengths, in queens and workers.

Our results stand in interesting partial contrast to those from honey bee queens (Babis et al. 2014; Richard et al. 2011). There, hydrocarbons showed little change in the Dufour’s gland, and age-related decreases in average chain length on the cuticle were driven by decreases in 27- and 29-carbon alkenes, as in our work. Interestingly, however, 27- and 29-carbon alkanes changed in the opposite direction, and insemination increased rather than decreased the average chain length.

Taken together, these findings suggest that hydrocarbons chain length might be regulated by conserved mechanisms probably tied to reproductive physiology with specific chain lengths generated by distinct sets of enzymes which are, in turn, activated by specific physiological events. The results are particularly interesting in the context of signal honesty, as the tight coupling between chemical signals and physiological processes supports the idea that some signals are inherently honest. Signal honesty is particularly important in social insects, where queens and workers have conflicting reproductive interests: queens benefit from inhibiting worker reproduction through their signals, whereas workers benefit from responding to these signals only when they reliably reflect the queen’s reproductive status. If chemical signals are constrained by the physiological mechanisms that produce them, they may be difficult or impossible to fake, limiting the potential for cheating. This idea, however, warrants further investigation.

Another common pattern in our study is the decrease in the alkene-to-alkane ratio with age in founder queens occurring in the Dufour’s and the labial glands and, to a small degree in the mandibular glands. A similar pattern was observed in the cuticular hydrocarbons of unmated honey bee queens (Babis et al. 2014). While a decrease in alkene-to-alkane ratio is also regulated by ovarian hormones in the housefly (Adams et al. 1984), we know little about the regulation of hydrocarbon desaturation in Hymenoptera. In honey bee workers, a chain-length-dependent change in cuticular alkenes, but not alkanes, was observed following an immune challenge (Richard et al. 2008) but no regulatory pathways producing this change were proposed. Further work is needed to understand what physiological mechanisms drive hydrocarbon desaturation in Hymenoptera. Importantly, in our study both metrics related to hydrocarbons – average chain length and alkene-to-alkane ratio – showed patterns shared by two or more different exocrine sources. These findings suggest that hydrocarbon components in different secretions, or at least their precursors, share a common biosynthetic source, most likely the fat body, and existing discrepancies between different sites are likely explained by differences in transport and uptake.

Unlike hydrocarbons, functionalized compounds such as esters, alcohols and terpenoids in our study showed large differences between secretions alongside some overlap in the patterns of change. Notably, the amounts of wax esters followed starkly opposite trends, decreasing with age and reproduction in the Dufour’s gland and increasing in the labial glands. At the same time, the proportions of cuticular acetate esters peaked in young founders but were low in both unmated queens and old founders. The contrast between the Dufour’s and labial glands was driven by two different processes: the disappearance of long-chain esters in the former and the increase in short-chain ones in the latter. This agrees with previous findings from *Bombus* (Derstine et al. 2021; Orlova et al. 2022) showing opposite trends in ester composition of the two glands with increasing fertility and forms an interesting contrast with previous work in the honey bee where short-chain esters in the Dufour’s gland decreased and some of the longer-chain ones increased after insemination. Terpenoids in our study overall decreased with mating in both the Dufour’s and labial glands, but the trend varied greatly between individual compounds. The overall decrease was driven by terpenoid esters of 16- and 18-carbon acids, the most abundant species dominating the terpenoid fraction. At the same time, unconjugated terpenes and terpenoid esters of 12- and 14-carbon acids showed great variability across the two glands. This is not surprising as both the Dufour’s and labial glands are likely to possess their own biosynthetic machinery for the biosynthesis of esters and terpenes. This biosynthesis is likely regulated by factors related to mating (Derstine et al. 2023, 2024; Katzav-Gozansky et al. 1997a).

Taken together, our findings offer a first glimpse into potentially shared patterns of chemical signal synthesis in a primitively eusocial bee. The contrasts that we observed with findings from the (relatively) closely related but highly derived honey bee and, conversely, the similarities with more distant and non-social Diptera highlight the need for comparative studies of chemical signaling incorporating diverse taxa with different social structures and exploring complete chemical profiles rather than isolated compounds or exocrine sites. The similar patterns observed across hydrocarbons, together with the distinct patterns of other functional groups, suggest that hydrocarbons are centrally produced and distributed among exocrine sites, whereas compounds such as esters and terpenes are likely synthesized de novo within their respective glands. Finally, our results show that changes in secretion occur simultaneously across multiple exocrine sites, suggesting crosstalk among them and highlighting the importance of studying individual exocrine sites within their broader chemical context. Together, these findings suggest that the combined chemical profile across multiple exocrine sites may convey more biologically relevant information than any single glandular secretion alone.

## Supplementary Information

Below is the link to the electronic supplementary material.

Supplementary Material 1 (XLSX 27.7 KB)

## Data Availability

The datasets generated and analyzed during the current study are available from the corresponding author upon reasonable request.
